# A randomized controlled trial evaluating the hemodynamic impact of ultrasound-guided great auricular nerve block in middle ear microsurgery

**DOI:** 10.1186/s12871-020-01155-y

**Published:** 2020-09-15

**Authors:** Jinsheng Liu, Kezhi Yuan, Hongling Zhou, Li Li, Guyan Wang, Tianzuo Li

**Affiliations:** 1 Department of Anesthesiology, Beijing Tongren Hospital, Capital Medical University, Beijing, China; 2 Department of Gastroenterology, Beijing Tongren Hospital, Capital Medical University, Beijing, China; 3 Department of Anesthesiology, Beijing Shijitan Hospital, Capital Medical University, Beijing, China

**Keywords:** Ultrasonography, Ear, middle, Nerve block, Hemodynamics

## Abstract

**Background:**

The peri-operative effectiveness of ultrasound-guided great auricular nerve block (GANB) in patients, especially in adult patients undergoing middle ear microsurgery remains unclear. We hypothesized that ultrasound-guided GANB would decrease the hemodynamic responsiveness to incision and opioid consumption in middle ear microsurgery as well as the post-operative analgesia requirement.

**Methods:**

Sixty patients undergoing middle ear microsurgery were randomized into two equal groups to receive either a GANB with 2 ml of 0.25% ropivacaine under ultrasound guidance (GANB group) or to receive a blank control intervention (without any performed injection) before general anesthesia inductions. The primary outcomes were hemodynamic changes of MAP (mean artery pressure) and HR (heart rate) to skin incision. The secondary endpoints were to determine the consumptions of propofol and remifentanil during the operation and the incidence of remedial analgesia 48 h post-operation to maintain VAS ≤ 3.

**Results:**

The MAP post incision in GANB group was significantly lower than that in control group (GANB group 93.83 ± 11.72 mmHg vs. control group 100.87 ± 12.65 mmHg, *P* = 0.029). The increases for MAP and HR post incision were also lower in GANB group (∆MAP GANB group 11.90 ± 8.32 mmHg vs. control group 19.83 ± 10.37 mmHg, *P* = 0.002; ∆HR GANB group 3.67 ± 5.30 beat min^− 1^ vs. control group 8.23 ± 8.56 beat min^− 1^, *P* = 0.016). Remifentanil consumption was significantly decreased in GANB group (GANB group 401.55 ± 100.51 μg h^− 1^ vs. control group 697.34 ± 215.45 μg h^− 1^, *P* = 0.000). The incidence of remedial analgesia post-operation in GANB group (5/30) was significantly lower than that in control group (20/30, *P* = 0.000).

**Conclusion:**

Ultrasound-guided GANB decreases the hemodynamic responsiveness to incision and remifentanil consumption in middle ear microsurgery as well as the post-operative analgesia requirement.

**Trial registration:**

This trial was retrospectively registered at http://www.chictr.org.cn with the registration number of ChiCTR1800014333 on 6 January, 2018.

## Background

Middle ear microsurgery is the most popular procedure in otology. The noxious stimulation from surgery can activate the sympathetic system, resulting in hypertension and increased HR, which are adversarial factors for improving the field quality and reducing hemorrhagic loss in middle ear microsurgery. Multifarious medications were applied in middle ear microsurgery to achieve controlled hypotension, better surgical field visibility or less blood loss [1–3]. Therefore, inhibition of hemodynamic response caused by noxious stimuli from the procedure as well as post-operative pain management are the most important tasks of anesthesia. Ultrasound-guided great auricular nerve block (GANB) is a potential solution to these problems. Ultrasound-guidance allows to visualize peripheral nerve block. It is more convenient and feasible to safely block a single distal nerve with a small dosage of local anesthetic. Thus, Ultrasound-guidance has the advantages of precise blocked area, accurate analgesic effect, and reduced side effects [4–6]. Scalp nerve block can blunt hemodynamic response to incision or get better hemodynamics in some neurosurgeries [7, 8]. However, the nerve innervation of postauricular incision area in middle ear microsurgery mainly but may not only derive from great auricular nerve [9]. In addition, the evaluation of ultrasound-guided GANB is mainly focused on volunteers and the literatures for GANB in middle ear microsurgery are derived from pediatric patients rather than adults [10–12]. Therefore, it is necessary to make a clinical evaluation on ultrasound-guided GANB in adult patients during the perioperative period of middle ear microsurgery. We conducted this study to examine the hypothesis that ultrasound-guided GANB could decrease the hemodynamic responsiveness to incision and opioid consumption in middle ear microsurgery as well as the post-operative analgesia requirement.

## Methods

### Design and patients

This prospective, randomized, parallel control trial was approved by the local Ethics Committee of Beijing Tongren Hospital on 20 June, 2016. (No. TRECKY2016–015). The study adhered to the CONSORT guidelines and was conducted in Beijing Tongren Hospital from November 2016 to March 2017. Written informed consent was obtained from each patient. Inclusion criteria were ASA I-II patients aged 18–60 years with otitis media, cholesteatoma or tympanic membrane perforations, scheduled for middle ear microsurgery with postauricular incisions. Exclusion criteria were coagulopathies, peripheral neuropathy, known allergy or hypersensitivity to ropivacaine, grade 2 or more serious hypertension (SBP values ≥160 mmHg and/or DBP values ≥100 mmHg), ischemic diseases of brain and heart, and clinically relevant abnormalities in electrocardiogram.

### Randomization and allocation concealment

Patients were randomly allocated into control group and GANB group with a ratio of 1:1 using a computer-generated list of random numbers. The allocation sequence was concealed from the researchers who enrolling and assessing participants in sequentially numbered, opaque, sealed and stapled envelopes. Aluminium foil inside the envelope was used to render the envelope impermeable to intense light. Corresponding envelopes were opened only after the participants came into the operating room. Blinding method was not used in this study due to the effectiveness assessing of GANB.

### Technique and anesthetic procedure

Both groups did not receive premedication. The electrocardiogram, heart rate, non-invasive blood pressure, SPO_2_ and BIS (Bispectral index) values were monitored in the study.

**Fig. 1:**
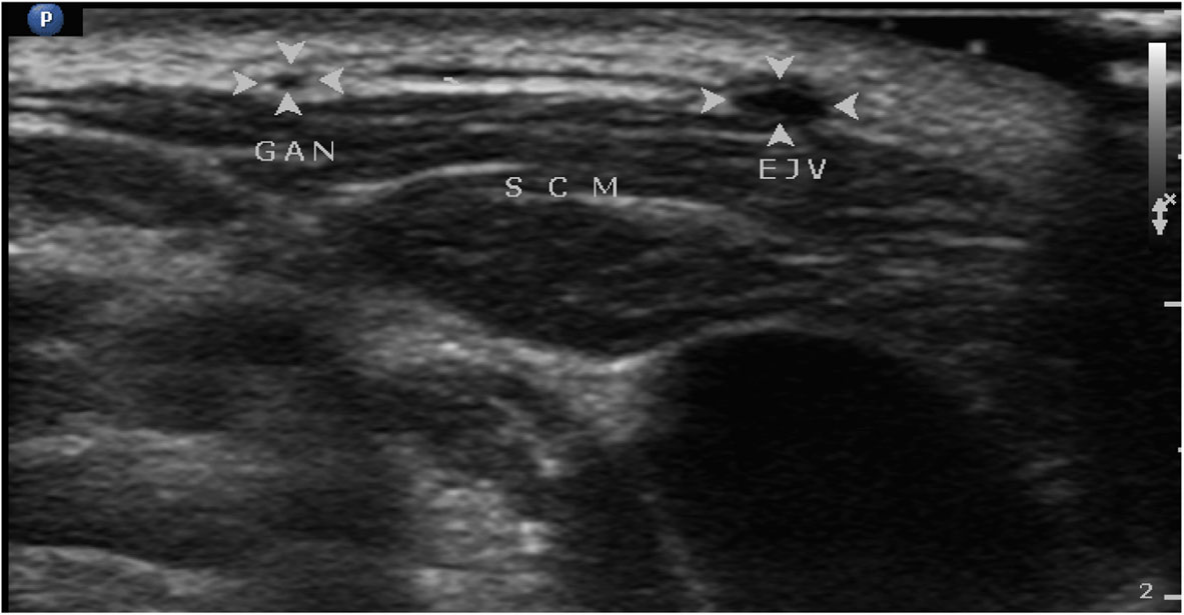
Cross sectional ultrasound view of the great auricular nerve (GAN, indicated by the arrows) at the surface of sternocleidomastoid muscle (SCM). EJV: external jugular vein. Right side = medial

Patients in GANB group received GANBs under ultrasound guidance before general anesthesia inductions according to the method described in Thallaj’s study [10] and our clinical practice. There was no any performed injection for patients in control group. The GAN (great auricular nerve) was examined with ultrasound equipment (Philips CX50 Diagnostic Ultrasound System) and a L12–3 MHz linear array transducer. The GAN was identified as a round or oval hypoechoic structure superficial to the sternocleidomastoid muscle (SCM) when the ultrasound transducer was horizontally put on the surface of SCM above the midpoint of its posterior border. GAN lied between external jugular vein (EJV) and the dorsal border of SCM (Fig. 1).

**Fig. 2:**
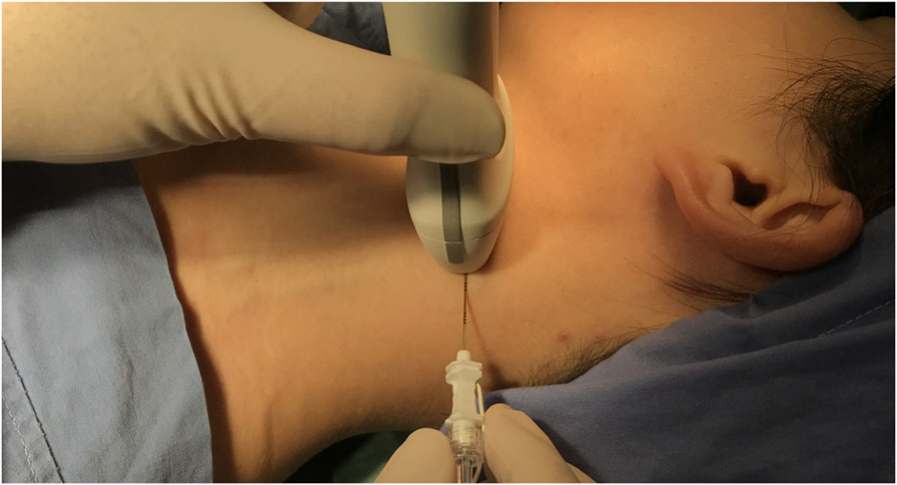
A simulation of in-plane needle ultrasound-guidance technique for GANB

The GAN was blocked with 2 ml ropivacaine (AstraZeneca Corporation, batch number: NAML, diluted by 0.9% saline) with a concentration of 0.25% in order to achieve a highly selective and completely blockade. An in-plane needle guidance technique was performed in all cases in GANB group (Fig. 2). All blocks were performed by the same anesthetist.

All patients in both groups were induced by midazolam 0.03 mg kg^−1^, propofol 1 mg kg^−1^, etomidate 0.2 mg kg^−1^, cisatracurium 0.2 mg kg^−1^, and then remifentanil was infused by plasma target-controlled infusion with Minto model at a concentration of 1 ng ml^−1^. Propofol also started to infuse along with remifentanil at a speed of 8 mg kg^− 1^ h^− 1^. A flexible laryngeal mask airway was used to facilitate mechanical ventilation to maintain EtCO_2_ 35 ~ 45 mmHg. General anesthesia was maintained by TIVA (total intravenous anesthesia) during the operation. Propofol was infused by a relatively stable speed of 6 ~ 8 mg kg^− 1^ h^− 1^ to keep a BIS value of 40 ~ 60. The cp_remi_ (target-controlled plasma concentration of remifentanil) was adjusted and maintained at 0.5 ng ml^− 1^ after intubation of laryngeal mask airway until incision was performed. After incision, the cp_remi_ was adjusted to 3 ng ml^− 1^, and then to maintain a reasonable level (setting range of cp_remi_: 1 ~ 10 ng ml^− 1^, with an increase or decrease step of 1 ng ml^− 1^ at an interval of 2 min) to make an ideal systolic blood pressure of 90 ~ 130 mmHg and an ideal HR of 50 ~ 90 beat min^− 1^ to meet the procedure demand until there was no ascending or descending tendency. This cp_remi_ level as an optimal concentration was sustained until the procedure was finished. Infusion of propofol and remifentanil were stopped simultaneously when the procedure was finished. No urethral catheterization was used in both groups during the procedure and in post anesthesia care unit.

All patients were followed up for 48 h post operation. Sensory regain was evaluated by pinprick test of bilateral mandibular angle area. The postoperative pain was evaluated by VAS (Visual Analog Scale), 50 mg of flurbiprofen axetil or 5 μg of sufentanil was infused intravenously as the remedial analgesia measure to maintain VAS ≤ 3.

### Measurement

Sensory loss or weaken to pinprick was assessed and compared with the contralateral area 10 min after the injection of ropivacaine in GANB group. The following areas were examined to assess the success of GANB: postauricular incision area, lobule and mandibular angle. The hemodynamic parameters (SBP, DBP, MAP, HR) and their difference between pre-incision and 1 min post-incision (i.e, ∆SBP, ∆DBP, ∆MAP, ∆HR) were employed as the primary outcome. The consumption of propofol and remifentanil, as well as the incidence of remedial analgesia 48 h post-operation were secondary outcome measures.

### Sample size calculation

In our pilot study, ∆MAP post incision in control group and GANB group was19 ± 10 mmHg and 12 ± 8 mmHg, respectively. By using a two-sided two-sample *t*-test along with 1-β = 0.8 and α = 0.05 in PASS 11 software, we got a sample size of *n*_1_ = *n*_2_ = 27. When a dropout rate of 10% was considered, a sample size of *n*_1_ = *n*_2_ = 30 was obtained.

### Statistical analysis

Data analysis was performed using SPSS Statistics 24. Data were expressed as mean ± SD. *Χ*^2^ test was used to analyze the composition ratio. Independent samples *t* test and paired samples *t* test were used to determine intergroup and intragroup difference, respectively. A *P* value< 0.05 was considered significant. The scatter dot plots and the VAS trend plot were drawn by GraphPad Prism version7.00.

## Results

### Basic information

**Fig. 3:**
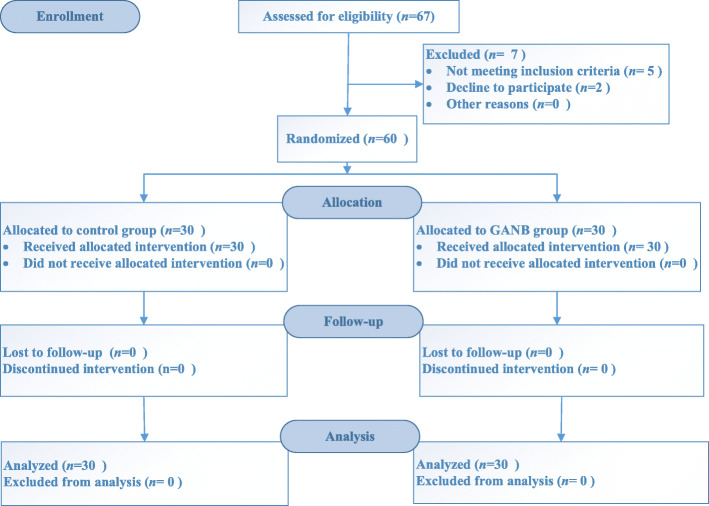
CONSORT flow diagram

Totally 67 patients were assessed for eligibility to participate in this study. Seven patients were excluded, including 5 patients who did not meet the inclusion criteria and 2 patients declined enrollment. The remaining 60 patients consented to participate the study. Thirty patients were allocated to GANB group and the other 30 patients were allocated to control group. All 60 patients completed the study without dropout or loss to follow up (Fig. 3). The age, height, weight, male: female ratio, BMI, and ASA classification were similar in both groups (Table 1).

**Table 1:** The demographic data

	Control group (*n* = 30)	GANB group (*n* = 30)	*P*
Age (yr)	40.30 ± 14.03	42.30 ± 12.15	0.557
Height (cm)	165.53 ± 7.64	166.03 ± 8.62	0.813
Weight (kg)	63.27 ± 10.99	64.43 ± 9.53	0.662
Male: female	16:14	15:15	0.796
BMI (kg m^−2^)	23.0 ± 3.4	23.4 ± 3.3	0.684
ASA class I:II	17:13	20:10	0.426

Data are presented as mean ± SD or number. BMI, body mass index

### Hemodynamic responsiveness on incision

**Fig. 4:**
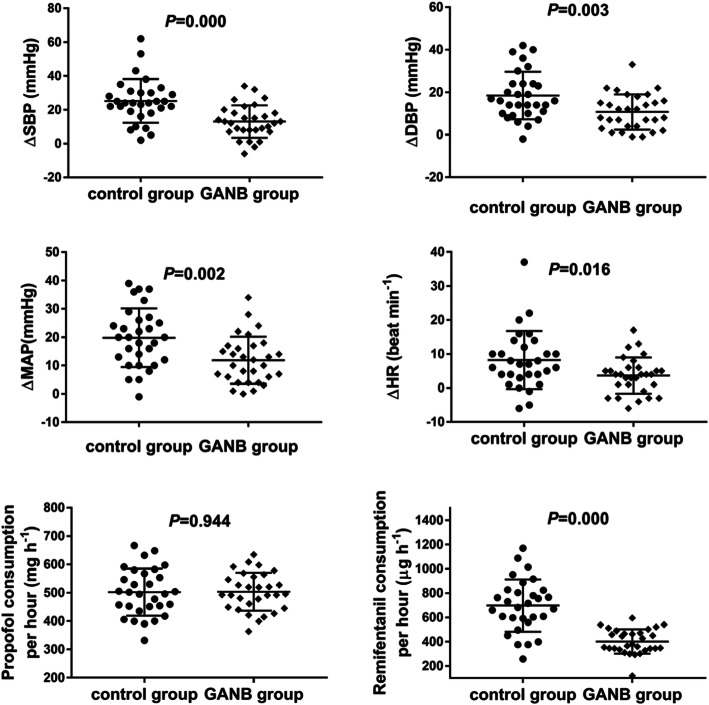
∆SBP, ∆DBP, ∆MAP and ∆HR pre-incision to post-incision, as well as consumptions of propofol and remifentanil per hour in control group and GANB group

The hemodynamic data and BIS values, as well as their increments pre-incision to 1 min post-incision in both groups were shown in Table 2. The SBP, DBP, MAP, HR and BIS values before incision were similar in control group and GANB group. All these indices had a significant increase 1 min post-incision. However, DBP and MAP, as well as the intercepts (∆SBP, ∆DBP, ∆MAP, ∆HR) in GANB group were significantly lower than those in control group (also shown in Fig. 4).

**Table 2:** The pre-incision and 1 min post-incision hemodynamic data and BIS values, as well as their increments

		Control group (*n* = 30)	GANB group (*n* = 30)	*P*
SBP (mmHg)	pre-incision	104.87 ± 9.14	109.57 ± 14.07	0.130
	post-incision	130.07 ± 16.64	122.60 ± 14.65	0.070
	∆SBP	25.20 ± 12.91	13.03 ± 9.60	0.000
DBP (mmHg)	pre-incision	68.53 ± 7.64	70.20 ± 8.47	0.427
	post-incision	87.00 ± 11.92	77.60 ± 17.26	0.017
	∆DBP	18.47 ± 11.15	10.73 ± 8.29	0.003
(mmHg)	pre-incision	81.03 ± 8.28	81.93 ± 9.71	0.701
	post-incision	100.87 ± 12.65	93.83 ± 11.72	0.029
	∆MAP	19.83 ± 10.37	11.90 ± 8.32	0.002
HR (beat min^−1^)	pre-incision	77.40 ± 12.93	79.33 ± 9.47	0.511
	post-incision	85.63 ± 12.86	83.00 ± 9.56	0.372
	∆HR	8.23 ± 8.56	3.67 ± 5.30	0.016
BIS	pre-incision	41.63 ± 7.20	38.67 ± 6.68	0.103
	post-incision	44.40 ± 8.36	40.50 ± 7.14	0.057
	∆BIS	2.77 ± 4.54	1.83 ± 3.02	0.352

Data are presented as mean ± SD

### General anesthetics consumption during the procedures

The duration of anesthesia, infusion duration of propofol and remifentanil, total consumption of propofol and propofol dosage per hour in both groups were similar. There was a significant decrease on remifentanil total consumption and remifentanil per hour consumption in GANB group (Table 3 and Fig. 4).

**Table 3:** General anesthetics consumption in the operations

	Control group	GANB group	
	(*n* = 30)	(*n* = 30)	*P*
Duration of anesthesia (h)	2.20 ± 0.64	2.48 ± 0.85	0.155
Infusion duration of propofol and remifentanil(h)	2.16 ± 0.64	2.44 ± 0.85	0.151
Total consumption of propofol (mg)	1083.59 ± 373.71	1241.39 ± 501.94	0.173
Total consumption of remifentanil (μg)	1511.56 ± 676.04	999.93 ± 472.09	0.001
Propofol consumption per hour (mg/h)	502.18 ± 83.16	503.56 ± 66.99	0.944
Remifentanil consumption per hour (μg/h)	697.34 ± 215.45	401.55 ± 100.51	0.000

Data are presented as mean ± SD

### The post-operative analgesia requirement

**Fig. 5:**
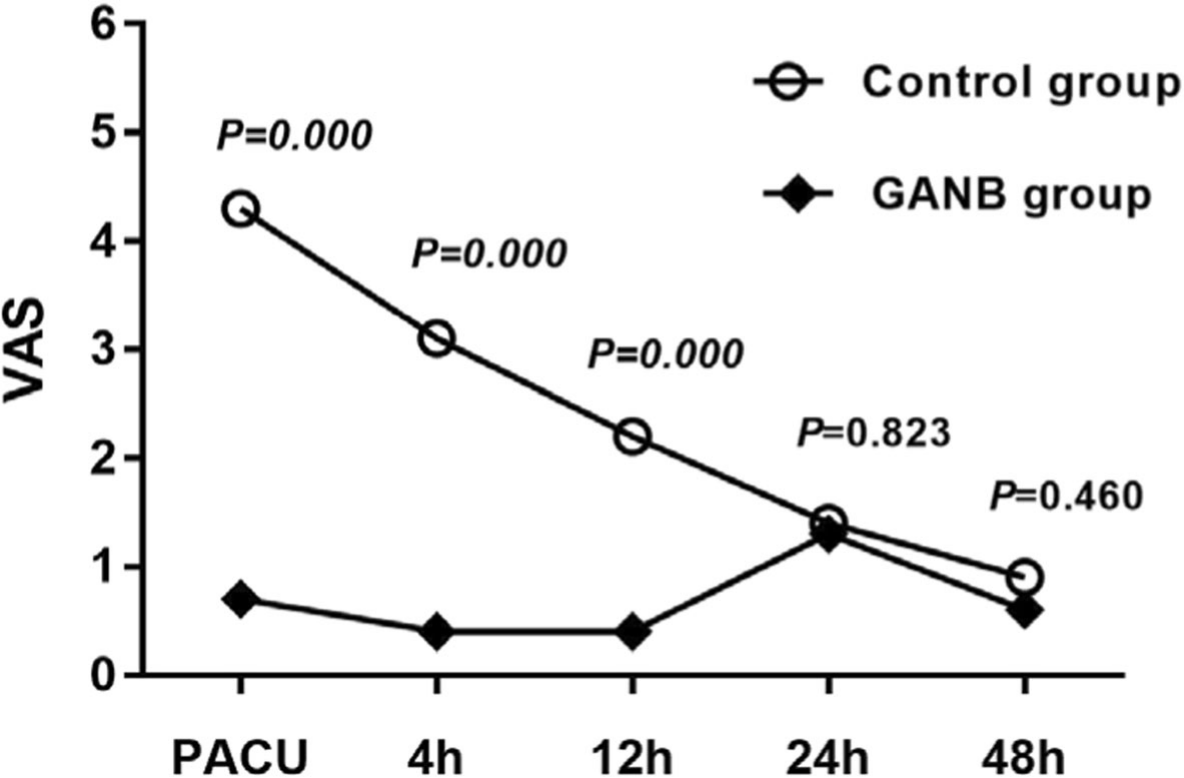
The VAS trend in PACU, and at 4, 12, 24, 48 h post operation

The postoperative pain was evaluated by VAS (Visual Analog Scale) for 48 h post-operation. The VAS trend in PACU (post operation care unit), and at 4, 12, 24, 48 h post operation were shown in Fig. 5. The VAS in PACU, at 4 h and 12 h were significantly lower in GANB group. Flurbiprofen axetil or sufentanil was infused intravenously as the remedial analgesia measure to maintain VAS ≤ 3. The incidence of remedial analgesia in GANB group was significantly lower than that in control group (GANB group 5:30 or 16.7% vs. control group 20:30 or 66.7%, *P* = 0.000).

## Discussion

Acute high blood pressure and increased HR are risk factors of hemorrhage in the operative field during middle ear microsurgery. Hypertension and increased HR may lead to more blood loss and bad field quality, and eventually affect the operative outcome. The blood pressure should be stable and on a relatively lower but safe level to reduce intraoperative bleeding and improve the visibility of the operative field [3, 13]. Noxious stimuli from incision may raise blood pressure and increase heart rate, which negatively affects the procedure. The hemodynamic changes resulted from incision were often regarded as a direct and effective index in the study of noxious stimuli and anesthesia [14, 15]. Peripheral nerve block is an effective method to minimize pain and hypertensive condition [7, 8]. In this study, the incision response of blood pressure and HR was investigated to evaluate whether GANB could suppress the response in middle ear microsurgery. The SBP, DBP, MAP, HR and BIS values before incision were similar in control group and GANB group, suggesting that GANB itself had no impact on the patient’s hemodynamics prior to the surgical procedure. The DBP and MAP post-incision as well as the intercepts (∆SBP, ∆DBP, ∆MAP, ∆HR) in GANB group were significantly lower than those in control group, indicating that GANB can effectively blunt the hemodynamic response to incision.

In this study, the sedation levels monitored by BIS were similar. We titrated cp_remi_ to maintain similar hemodynamics in both groups. It was found that the general anesthesia duration and the consumption of propofol were similar in both control group and GANB group. However, the total and per hour consumption of remifentanil in GANB group were significantly lower than that in control group. Remifentanil is a good measure for controlled hypotension in middle ear surgery [16, 17]. The sparing effect of peripheral nerve block on the intraoperative consumption of remifentanil or other kind of opioids has been confirmed by many literatures [18–20]. The decrease of remifentanil consumption also indicated that GANB can provide adequate analgesia in middle ear microsurgery.

In this study, endotracheal tube was replaced by flexible laryngeal mask airway and no urinary catheter was used. The throat or oral pain from the endotracheal tube and the irritation from urinary catheters were reduced to minimum. In such a condition, the post-operation pain was mainly from surgical trauma. The post-operative VAS of pain in PACU, at 4 h and 12 h were significantly lower in GANB group. Meanwhile, the incidence of remedial analgesia in GANB group (5:30) was significantly lower than control group (20:30, *P* = 0.000). The durations of GANB observed in this study in GANB group was 11.7 ± 1.9 h, which may contribute to the lower incidence of moderate or severe pain in GANB group. Therefore, when the effect of GANB ended, we found a slight rebound of pain in control group at 24 h post operation, although it was commonly a mild pain. This suggested that GANB was effective for post-operative analgesia of middle ear microsurgery in adult patients, which was consistent with the results from previous literatures, wherein a single GAN block can provide complete analgesia in nearly half the patients and decrease the overall incidence of postoperative vomiting in children undergoing tympanomastoid surgery [12].

The opisthotic incision of mastoid area was used for every subject in this study, which was common in middle ear microsurgery. The nerve innervation of this area mainly but may not only derive from GAN’s branch. The great auricular nerve (GAN) originates from the cervical plexus at the levels of C_2_ and C_3_, which is the largest sensory branch of superficial cervical plexus [21]. The applied anatomy of great auricular nerve is simple [22, 23], but the nerve supply of the auricle is complex, which comes from the innervation of GAN, auricular branch of vagus nerve, auriculotemporal nerve and lesser occipital nerve. In addition, double innervation and innervation variation may exist in the nerve innervation of different areas of auricle [9]. Despite the complexity of innervation, the results from this study suggest that GANB provides adequate analgesia effects for the opisthotic incision in middle ear microsurgery.

The conventional approach for GANB is a landmark-based technique or achieved by superficial cervical plexus block. The great auricular nerves were successfully blocked with ropivacaine under ultrasound guidance in all 30 patients in GANB group. In this study, ultrasound-guided in-plane technique was used for GANB. Ultrasound-guided GANB has the advantages of ultrasound-guided technology, which is more accurate and effective with significantly reduced dosage of local anesthetic, less complications and less influence on the other adjacent nerves [6, 19, 20]. GAN is shallow in position and easy to access, we didn’t find any neuropathic complication in this study. One case of local hematoma was found in our pilot study, but it didn’t appear in this study by increasing the press duration of the puncture point.

One limitation of our study was that there was no blind method because of the study design and ethical consideration, which may increase the possibility of bias on the result judgment. Nevertheless, the success and duration of GANB through sensory loss and regain test to pinprick provides a better indication of its effectiveness on incision reaction and post-operative analgesia. Another limitation was that we also found an increase on SBP, DBP, MAP and HR post-incision than that of pre-incision in GANB group, although less than that of the control group. This may be related to the complex innervation of auricle.

## Conclusion

In conclusion, ultrasound-guided GANB reduces the hemodynamic responsiveness as well as the opioid consumption of remifentanil in middle ear microsurgery in adult patients. Patients who received GANB can get a relief from postoperative pain.

## Data Availability

The datasets used during the current study are available from the corresponding author on reasonable request.

## Abbreviations

ASA: American society of anesthesiologists
GAN: Great auricular nerve
GANB: Great auricular nerve block
SBP: Systolic blood pressure
DBP: Diastolic blood pressure
MAP: Mean arterial pressure
